# CD4:CD8 lymphocyte ratio as a quantitative measure of immunologic health in HIV-1 infection: findings from an African cohort with prospective data

**DOI:** 10.3389/fmicb.2015.00670

**Published:** 2015-07-01

**Authors:** Jianming Tang, Xuelin Li, Matthew A. Price, Eduard J. Sanders, Omu Anzala, Etienne Karita, Anatoli Kamali, Shabir Lakhi, Susan Allen, Eric Hunter, Richard A. Kaslow, Jill Gilmour

**Affiliations:** ^1^Department of Medicine, University of Alabama at Birmingham, Birmingham, ALUSA; ^2^International AIDS Vaccine Initiative, New York, NYUSA; ^3^Department of Epidemiology & Biostatistics, University of California, San Francisco, San Francisco, CAUSA; ^4^Centre for Geographic Medicine Research, Kenya Medical Research Institute, KilifiKenya; ^5^Centre for Clinical Vaccinology and Tropical Medicine, University of Oxford, OxfordUK; ^6^Kenya AIDS Vaccine Initiative, NairobiKenya; ^7^Projet San Francisco, KigaliRwanda; ^8^Uganda Virus Research Unit on AIDS, Medical Research Council/Uganda Virus Research Institute, MasakaUganda; ^9^Zambia-Emory HIV Research Project, LusakaZambia; ^10^Department of Pathology and Laboratory Medicine, Emory University, Atlanta, GAUSA; ^11^Emory Vaccine Center, Emory University, Atlanta, GAUSA; ^12^Department of Epidemiology, University of Alabama at Birmingham, Birmingham, ALUSA; ^13^International AIDS Vaccine Initiative, Human Immunology Laboratory, Chelsea and Westminster Hospital, LondonUK

**Keywords:** Africa, CD4:CD8 ratio, HIV-1, subtype, HLA, statistical models, viral load

## Abstract

In individuals with human immunodeficiency virus type 1 (HIV-1) infection, CD4:CD8 lymphocyte ratio is often recognized as a quantitative outcome that reflects the critical role of both CD4^+^ and CD8^+^ T-cells in HIV-1 pathogenesis or disease progression. Our work aimed to first establish the dynamics and clinical relevance of CD4:CD8 ratio in a cohort of native Africans and then to examine its association with viral and host factors, including: (i) length of infection, (ii) demographics, (iii) HIV-1 viral load (VL), (iv) change in CD4^+^ T-lymphocyte count (CD4 slope), (v) HIV-1 subtype, and (vi) host genetics, especially human leukocyte antigen (HLA) variants. Data from 499 HIV-1 seroconverters with frequent (monthly to quarterly) follow-up revealed that CD4:CD8 ratio was stable in the first 3 years of infection, with a modest correlation with VL and CD4 slope. A relatively normal CD4:CD8 ratio (>1.0) in early infection was associated with a substantial delay in disease progression to severe immunodeficiency (<350 CD4 cells/μl), regardless of other correlates of HIV-1 pathogenesis (adjusted hazards ratio (HR) = 0.43, 95% confidence interval (CI) = 0.29-0.63, *P* < 0.0001). Low VL (<10,000 copies/ml) and HLA-A*74:01 were the main predictors of CD4:CD8 ratio >1.0, but HLA variants (e.g., HLA-B*57 and HLA-B*81) previously associated with VL and/or CD4 trajectories in eastern and southern Africans had no obvious impact on CD4:CD8 ratio. Collectively, these findings suggest that CD4:CD8 ratio is a robust measure of immunologic health with both clinical and epidemiological implications.

## Introduction

Progressive and systemic deterioration of immunologic health is a major hallmark of human immunodeficiency virus type 1 (HIV-1) pathogenesis (Taylor et al., 1989; Miedema, 1992; Brenchley et al., 2006; Maartens et al., 2014). The status of immunologic health is routinely assessed by several quantitative traits that center on CD4^+^ T-cells (CD4), including absolute CD4 count (cells/μl), CD4 percentage, change in CD4 count over time (CD4 slope), and/or two thresholds of severe CD4 deficiency (typically <350 and <200 cells/μl). These CD4-based and often partially correlated outcomes have also been used for studying quantitative trait loci (QTLs) in viral and host genomes (Lazaryan et al., 2011; Apps et al., 2013; Bartha et al., 2013; Peterson et al., 2013), with clear evidence that determinants of CD4-related manifestations of HIV-1 infection have rather limited overlap with those of either virologic measures (Lazaryan et al., 2011; Amornkul et al., 2013; Antoni et al., 2013; Peterson et al., 2013; Prentice et al., 2013, 2014b) or those of HIV-1 acquisition (Tang et al., 2008; Gao et al., 2010; Song et al., 2011; Merino et al., 2012). As CD4 data are sparse in many resource-poor regions, current understanding of immunologic health in HIV-1-infected Africans is still limited. Recent observation of a low threshold CD4 count (as few as 457 cells/μl) in HIV-1 seronegative Africans (Karita et al., 2009) may pose further challenges for making guidelines and policies based solely on CD4 count thresholds.

Among other immunologic markers of HIV-1 pathogenesis, CD8 activation (Giorgi et al., 1999; Sousa et al., 2002), CD8 exhaustion (Eichbaum, 2011; Hinrichs et al., 2011), CD4:CD8 ratio (Taylor et al., 1989; Zaman et al., 2000; Margolick et al., 2006; Pahwa et al., 2008), and delayed-type hypersensitivity (DTH) to recall antigens (Dolan et al., 2007) can also serve as outcome measures that reflect immunologic health (or lack of). Documentation of CD4:CD8 ratio as a genetically controlled trait in healthy humans (Amadori et al., 1995; Ferreira et al., 2010) implies that factors associated with CD4:CD8 ratio may offer novel insights about the wide spectrum of HIV-1-related immune malfunction. The CD4:CD8 ratio is rarely measured below 1.0 in healthy subjects (Amadori et al., 1995), so an inverted CD4:CD8 ratio is often viewed as clinically relevant (Zaman et al., 2000; Pahwa et al., 2008). Examination of CD4:CD8 ratio as another quantitative trait can be important to patient care, especially when T-cell immunophenotyping using banked or newly collected samples becomes increasingly feasible (Sambor et al., 2014). Accordingly, our main objective was to characterize the relationships between CD4:CD8 ratio and HIV-1 disease outcomes in an African cohort with sufficient follow-up data.

## Materials and Methods

### Study Population

This study focused on native Africans who were recent HIV-1 seroconverters (SCs) enrolled from Kenya, Rwanda, Uganda, and Zambia under a uniform study protocol developed and implemented by the International AIDS Vaccine Initiative (IAVI; Price et al., 2011; Amornkul et al., 2013). All volunteers underwent written informed consent procedures prior to study-related procedures that were approved annually by institutional review boards at all collaborating institutions.

### Follow-Up Strategies before and after HIV-1 Infection

Identification of SCs relied on frequent (monthly to quarterly) testing of HIV-1 seronegative subjects at high risk of acquiring HIV-1 infection through heterosexual and homosexual exposure, with the vast majority being partners of HIV-1 discordant, heterosexual couples and/or individuals diagnosed with sexually transmitted infections. As described in detail elsewhere (Karita et al., 2007; Amornkul et al., 2013; Prentice et al., 2013), the estimated date of HIV-1 infection (EDI) for each subject was defined as one of the following: (i) the midpoint between the last seronegative and first positive HIV-1 antibody tests, (ii) 2 weeks before the first positive test for HIV-1 p24 antigen in plasma, (iii) 10 days before the first positive test for plasma viral load (VL) while being negative for both p24 and rapid HIV-1 antibody tests, and (iv) event date for the only known high-risk exposure. Following confirmation of HIV-1 infection (detection of VL), clinical visits were scheduled monthly for the first 3 months after EDI, quarterly for the 3–24 months interval, and every 6 months thereafter. Initiation of antiretroviral therapy (ART) followed national guidelines (Ngongo et al., 2012), and all visits and VL measurements after ART initiation were excluded. In all, 499 SCs (Supplementary Table S1) were selected based on availability of biological specimens for DNA extraction and human leukocyte antigen (HLA) class I genotyping, as well as at least three time points of VL in the early chronic phase (3–24 months) of infection, with no gap greater than 1 year between two consecutive VL measurements. The SCs excluded from analyses (*n* = 81) were mostly those with limited follow-up (less than three eligible visits for various outcome measures) or lack of biological specimens.

### Quantification of HIV-1 Viral Load (VL)

Plasma VL (HIV-1 RNA copies/ml) was measured at a central location (Clinical Laboratory Services, Johannesburg, South Africa) using the Amplicor Monitor v1.5 assay (Roche Applied Science, Indianapolis, IN, USA) through January 2011 and the Abbott real-time HIV-1 v1.0 assay (Abbott Laboratories, Abbott Park, IL, USA) thereafter and following good clinical laboratory practices (Amornkul et al., 2013). Eligible VLs in the 3- to 24-month interval were all beyond the acute-phase of infection (Tang et al., 2011). The geometric mean VL (Prentice et al., 2014b) was calculated from the average log_10_ VL during the 3- to 24-month interval and then divided into three categories (Fideli et al., 2001; Tang et al., 2010) with biological and epidemiological implications: low (<4.0 log_10_), medium (4.0–5.0 log_10_), and high (>5.0 log_10_). For log_10_-transformation, all VLs below the lower limit of detection (400 RNA copies/mL) were assumed to be 1.30 (half of log_10_ 400), as other alternatives (e.g., 2.30 log_10_ or 200 copies/ml) yielded similar results in data analyses (Prentice et al., 2014b).

### Viral Sequencing and Human Leukocyte Antigen (HLA) Class I Genotyping

Methods for HIV-1 *pol* gene sequencing and subtype determination have been described elsewhere (Tang et al., 2011; Amornkul et al., 2013; Prentice et al., 2014b). PCR-based assays also resolved allelic variants at three HLA class I genes (*HLA-A*, *HLA-B*, and *HLA-C*; Tang et al., 2010; Merino et al., 2012; Prentice et al., 2014b). Assignment of HLA haplotypes followed algorithms described elsewhere (Tang et al., 2010; Prentice et al., 2013, 2014b).

### Immunologic Outcomes and Immunodeficiency in the Absence of Antiretroviral Therapy

For our study population, CD4 count was the initial outcome defined by T-cell immunophenotyping (Amornkul et al., 2013; Prentice et al., 2013) performed at individual clinics using the FACScount System (Beckman Coulter Ltd., London, UK). These assays also quantified CD4:CD8 ratio. For consistency with previously applied criteria, we considered CD4:CD8 ratio >1.0 as an indication of immunologic health (lack of disease progression). The date of the first of two consecutive visits with CD4 count <350 cells/μL was deemed the onset of severe immunodeficiency (Amornkul et al., 2013).

### Descriptive Statistics

With a focus on data beyond the acute phase (first 3 months) of HIV-1 infection, subjects with contrasting CD4:CD8 ratios (>1.0 versus ≤1.0) during the 3- to 24-month period after EDI were compared for their overall baseline characteristics, including *t*-test for quantitative variables with a normal distribution, Wilcoxon’s rank-sum test for quantitative variables lacking a normal distribution, and χ^2^ or Fisher exact test for categorical variables (Supplementary Table S1). These and other analytical procedures were done using SAS, version 9.3 (SAS Institute, Cary, NC, USA). All baseline characteristics that differed between two major patient groups (CD4:CD8 ratio >1.0 versus ≤1.0) were treated as covariates in subsequent analyses. The inclusion of data over the 24- to 36-month period after EDI (not applicable to all subjects) led to similar conclusions.

### Central Hypothesis and Analytical Procedures

This study aimed to test a central hypothesis that CD4:CD8 ratio as a composite outcome is distinct from two conventional measures (VL and CD4 slope) of HIV-1 pathogenesis, after accounting for potential confounders like geography (eastern and southern Africa), sex, and major viral subtypes (A1, C, and others). Statistical analyses focused on: (i) the dynamics of CD4:CD8 ratio, VL, and CD4 slope in the first 3 years after EDI; (ii) the pairwise relationships between CD4:CD8 ratio, VL, and CD4 slope; (iii) the prognostic value of early CD4:CD8 ratio for subsequent disease progression; and (iv) host and viral correlates of CD4:CD8 ratio. Main analytical procedures included the following: (a) local regression (LOESS) curves, (b) Spearman’s correlation test, (c) Kaplan–Meier curves and Cox proportional hazards models, and (d) logistic regression models. To maximize sample size, CD4:CD8 ratio from the 3- to 24-month interval was analyzed first. Alternative analyses considered the addition of data from the 24- to 36-month period (not applicable to all subjects). Summary statistics, including correlation coefficients (rho), regression beta (β), HR, odds ratio (OR), 95% CI, *P*-value, and false discovery rate (FDR or *q*-value) were tabulated using SAS, version 9.3, as described in earlier work related to the same cohort (Prentice et al., 2013, 2014b). The overall performance of multivariable logistic regression models was also assessed using the area under the curve (AUC) estimates (*C*-statistics).

### Refinement of Host Genetic Factors Based on Linkage Disequilibrium (LD) and Biological Relevance

Wherever possible, HLA factors showing putative associations with CD4:CD8 ratio were refined by analyses of LD profiles and HLA haplotypes in subjects before and after stratification by geography (eastern versus southern Africa), with further reference to fully resolved haplotypes seen in other populations (Cao et al., 2001). Alternative analyses of 2- and 3-locus HLA haplotypes were deemed informative if the adjusted effect sizes improved over those for the component alleles. The likelihood of biological relevance was evaluated in the context of (i) HIV-1-specific CTL epitopes and escape mutations documented for individual HLA allelic products^1^^,^^2^ (Carlson et al., 2014), (ii) relationships to single nucleotide polymorphisms (SNPs) that have biological and/or epidemiological importance (Horton et al., 2004; Fellay et al., 2009; Prentice et al., 2014a), and (iii) other evidence as reported in the literature, especially the Finemapping Data Portal^3^ (Farh et al., 2015) and the HaploReg database^4^ (last accessed in April 2015).

## Results

### The Dynamics of CD4:CD8 Ratio in Primary HIV-1 Infection

**FIGURE 1:**
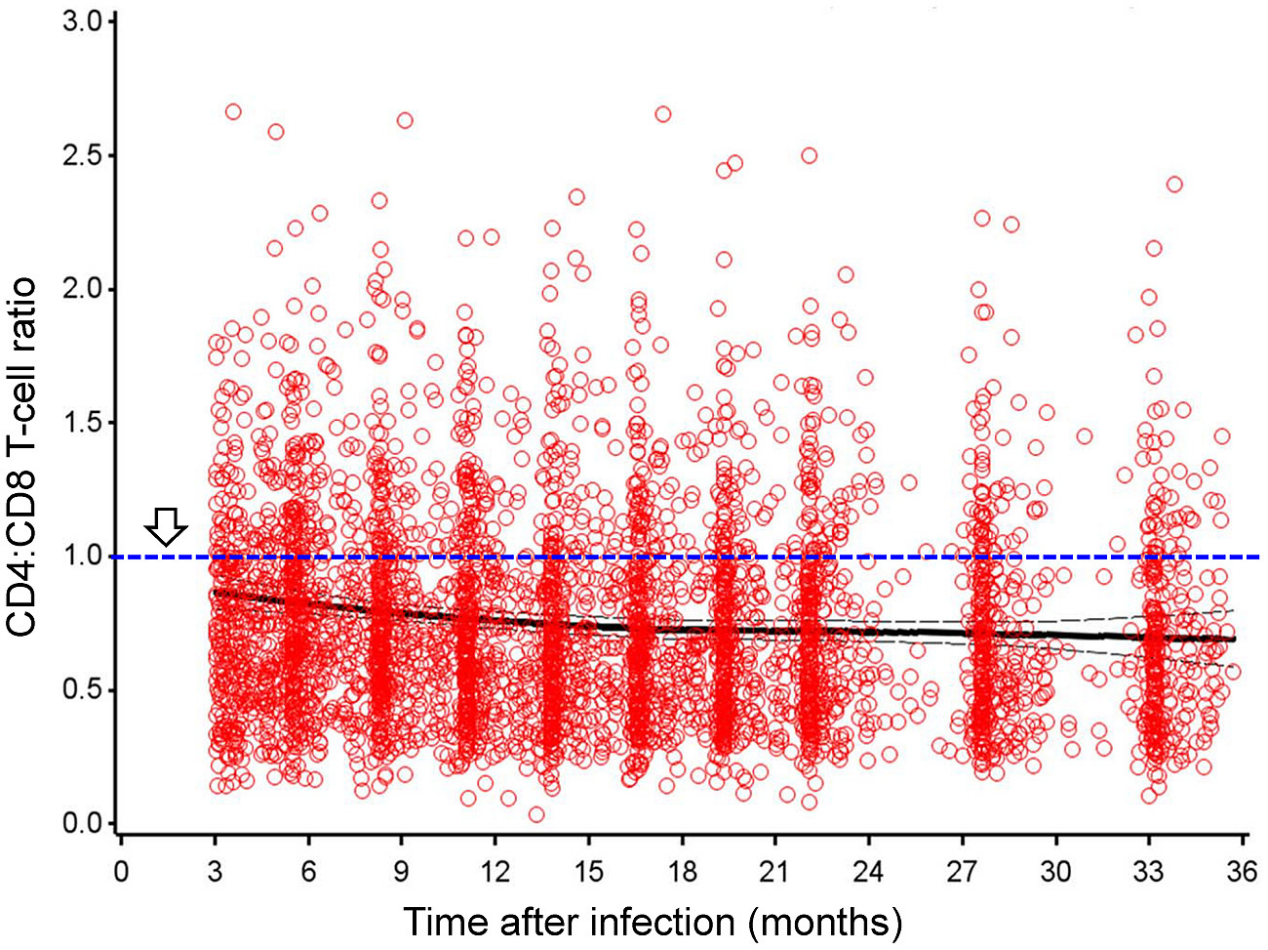
**CD4:CD8 T-lymphocyte ratio in early human immunodeficiency virus type 1 (HIV-1) infection, in the absence of antiretroviral therapy.** Results are shown for 499 seroconverters (SCs; native Africans) with 4,144 person-visits during the 3- to 36-month period after estimated date of infection. Thick and thin lines correspond to the mean ratio and 95% confidence interval (CI), respectively. Arrow points to the threshold of abnormal (inverted) ratio that is rarely seen in HIV-1 seronegative, healthy subjects.

Among native African subjects enrolled between February 2006 and December 2011, 196 Zambians, 125 Ugandans, 102 Kenyans, and 76 Rwandans had at least three measurements of CD4:CD8 ratio and other outcomes (VL and CD4 slope) within the 3- to 24-month intervals after EDI. Overall, CD4:CD8 ratio was steady during this early period of infection (**Figure 1**), with a heavy bias toward the ≤1.0 (abnormal) group (*P* = 2.2 × 10^-16^ in normality tests). For seven consecutive sliding time windows (3 months each), the pairwise Spearman rho values for comparing cross-sectional CD4:CD8 ratio ranged from 0.71 to 0.88 (*P* < 0.0001 for all; **Table 1**). The mean CD4:CD8 ratio within the 3- to 24-month intervals had a modest, inverse correlation with geometric mean VL (Spearman rho = -0.33, *P* < 0.0001), accompanied by a weak, positive correlation with CD4 slope (Spearman rho = 0.14, *P* < 0.01; **Table 2**). A negative correlation between geometric mean VL and CD4 slope was weak as well (rho = -0.20, *P* < 0.0001), suggesting that these three outcome measures were mostly independent of one another. Similar results were observed when additional data from the 24- to 36-month period were included in the correlation analyses (**Figure 1** and **Table 2**).

**Table 1:** Pairwise Spearman’s correlation coefficients (rho) for cross-sectional CD4:CD8 ratio measurements (seven consecutive time windows in early human immunodeficiency virus type 1 (HIV-1) infection).

Time window (subjects)^a^	3–6	6–9	9–12	12–15	15–18	18–21	21-24
(a) 3–6 months (424)	**1.00**						
(b) 6–9 months (446)	0.81	**1.00**					
(c) 9–12 months (458)	0.78	0.83	**1.00**				
(d) 12–15 months (434)	0.78	0.82	0.88	**1.00**			
(e) 15–18 months (425)	0.75	0.80	0.87	0.88	**1.00**		
(f) 18–21 months (400)	0.71	0.76	0.83	0.84	0.86	**1.00**	
(g) 21–24 months (375)	0.72	0.74	0.82	0.83	0.84	0.86	**1.00**

*^a^The timing for measuring CD4:CD8 ratio in 499 subjects is not always equally spaced, as precision in inferring dates of infection can vary by the three methods used (see Materials and Methods). *P* < 0.0001 in all tests. The maximum value under each time window is shown in bold.*

**Table 2:** Spearman correlation coefficients for mean CD4:CD8 ratio, set-point viral load (VL), and CD4 slope in 499 HIV-1 seroconverters (SCs).

	3–24 months after EDI^a^			3–36 months after EDI^a^		
Outcomes	CD4:CD8 ratio	VL	CD4 slope	CD4:CD8 ratio	VL	CD4 slope
CD4:CD8 ratio (mean)	**1.00**			**1.00**		
VL (geometric mean)	-0.33	**1.00**		-0.33	**1.00**	
CD4 slope	0.14	-0.20	**1.00**	0.20	-0.25	**1.00**

*^a^Results are shown for two overlapping time intervals after estimated date of infection (EDI). The maximum value under each column is shown in bold.*

### The Prognostic Value of Early CD4:CD8 Ratio

**FIGURE 2:**
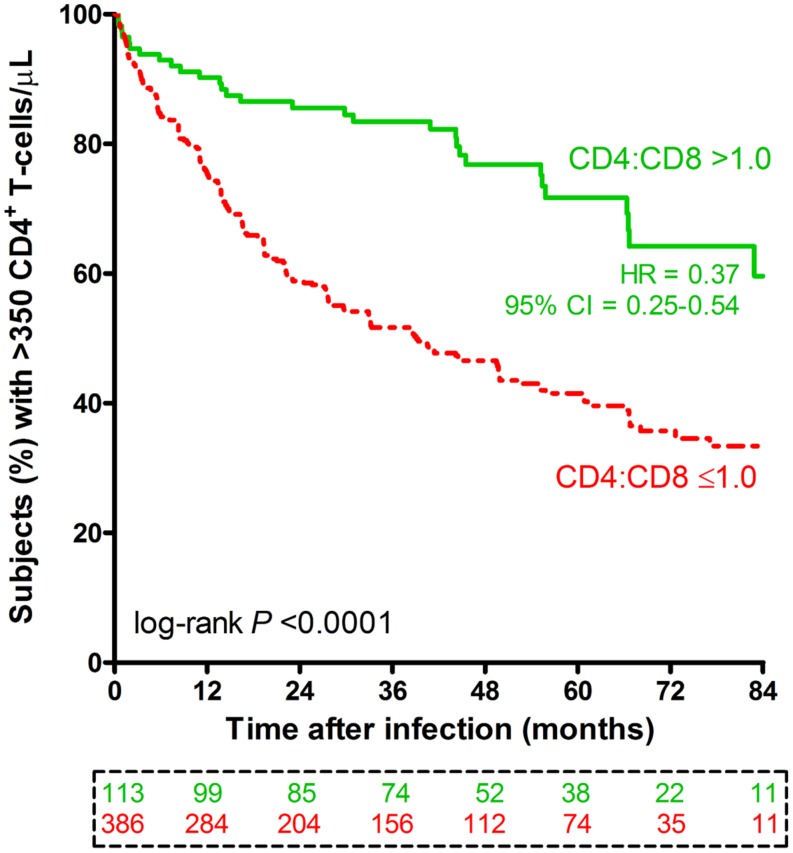
**Progression to severe immunodeficiency among 499 HIV-1 SCs stratified by early CD4:CD8 ratio.** Based on the first CD4:CD8 ratio measured beyond the initial 3-month period of infection, subjects are divided into two subgroups (CD4:CD8 ratio >1.0 in green color versus ≤1.0 in red color). The first of two consecutive visits with CD4^+^ T-cell count <350 cells/μL is plotted as the event time (Amornkul et al., 2013). The numbers of subjects available at nine time points are boxed and color coded. The crude hazard ratio (HR) and 95% CI are based on a Cox proportional hazards model (unadjusted). The adjusted model is shown in **Table 3**.

Beyond the initial 3-month period (acute phase) after EDI, the first available CD4:CD8 ratio was relatively normal (>1.0) in 113 (or 22.6%) subjects and abnormal (≤1.0) in the remainder (77.4%). The rates of subsequent disease progression, as measured by time to CD4 count <350 cells/μL, clearly differed (log-rank *P* < 0.0001) between these two immunologic subgroups (**Figure 2**). A favorable prognosis (a crude HR of 0.37, 95% CI = 0.25–0.54, *P* < 0.0001) for patients with CD4:CD8 ratio >1.0 was evident over a time span of up to 7 years after EDI. These HR estimates were insensitive to statistical adjustments for other potential confounders, including demographics, two reported HLA variants (HLA-B*45:01 and B*81:01), and geometric mean VL: the adjusted HR for CD4:CD8 ratio >1.0 was 0.43 (95% CI = 0.29–0.63, *P* < 0.0001; **Table 3**).

**Table 3:** Progression to severe immunodeficiency (CD4 count <350 cells/μL): prognosis based on early CD4:CD8 ratio and other potential factors in 499 HIV-1 seroconverters.

Factors in model (no. of subjects)	*n*	HR	95% confidence interval (CI)	*P*
Age > 40 years	75	1.78	1.29-2.45	<0.001
Age ≤ 40 years	424	1.00	–	–
Female sex	187	1.16	0.88–1.52	0.294
Male sex	312	1.00	–	–
Region: Zambia (southern Africa)	196	1.28	0.98–1.66	0.066
Region: eastern Africa	303	1.00	–	–
HLA-B*45:01^a^	81	1.38	1.01–1.90	0.043
HLA-B*81:01^a^	25	0.44	0.22–1.00	0.049
Low VL (<10,000 RNA copies/mL)	142	0.33	0.22–0.49	<0.0001
Medium VL (10,000–100,000)	265	1.00	–	–
High VL (>100,000)	92	1.67	1.23–2.25	<0.001
Early CD4:CD8 ratio^b^ >1.0	113	0.43	0.29–0.63	<0.0001
Early CD4:CD8 ratio^b^ ≤1.0	386	1.00	–	–

*^a^As reported previously for the study cohort (based on interim data analyses; Amornkul et al., 2013; Prentice et al., 2013).*

*^b^First available measurement beyond the acute phase of infection (see text).*

### Factors Associated with CD4:CD8 Ratio

In stepwise univariable models (Supplementary Table S1), patients defined by their average CD4:CD8 ratios (>1.0 and ≤1.0) during the 3- to 24-month period after EDI were highly comparable (*P* > 0.13 in all tests) in terms of age, sex ratio, and distribution of five HLA variants (B*18, B*45, B*53, B*57, and B*81) that were previously associated with VL and/or CD4 count in the same cohort (Amornkul et al., 2013; Prentice et al., 2013). The two immunologic subgroups did show clear differences in geography (*P* < 0.001) and HIV-1 subtype (*P* = 0.009). Similar results were seen when the time horizon for calculating the average CD4:CD8 ratio was expanded to the 3- to 36-month period after EDI (data not shown). Further analyses focused on the average CD4:CD8 ratios over the 3- to 24-month period alone.

### HLA-A*74:01 as a Novel Correlate of CD4:CD8 Ratio

Apart from hypothesis-testing for B*18, B*45, B*53, B*57, and B*81 (all with related evidence from earlier work), 29 other HLA class I variants were also frequent enough (≥5%) for association analyses. Based on regression models adjusted for age, sex, and geography, A*74:01 was most noteworthy for its favorable association with mean CD4:CD8 ratio >1.0 (OR = 2.29, *P* = 0.005, *q* = 0.172), while the remaining HLA class I variants were readily dismissed (*P* > 0.05 in all tests; Supplementary Table S2).

### HLA-A*74:01-Related Haplotypes

In the study population, HLA-A*74:01 was in weak LD (*r*^2^ < 0.05) with B*35:01, B*42 (*42:01 and *42:02), B*44 (*44:03 and *44:15), B*49:01, B*58 (*58:01 and *58:02), C*02:10, C*04:01, and C*17:01 (*P*-values ranging from 0.0001 to 0.042). The individual haplotypes defined by LD profile and random combination were too rare (<1.8% for all) to justify sub-analyses. Data stratification by country did not facilitate further analysis of HLA haplotypes either.

### Bioinformatic Analyses for HLA-A*74:01

The current HIV Molecular Immunology Database has compiled a total of 991 HIV-1 CTL epitope polymorphisms associated with *HLA-A* alleles, but none for HLA-A*74:01. Additional data specific for southern Africans (including Zambians; Carlson et al., 2014) indicate that HLA-A*74:01, as part of the A03 supertype, has been associated with three mutations in HIV-1 Gag (R20K/S, R91X, and V94I) and two in Pol (R432K and R521K). These mutations often overlap with the optimal epitope, GR11 in HIV-1 Gag-p24, for A*74:01 (Matthews et al., 2011). Meanwhile, an intergenic SNP (rs9468675 G/T, also known as rs114788707 or rs118104426) that effectively tags A*74:01 in an African population (Yoruban; de Bakker et al., 2006) has been mapped to an enhancer element (the HaploReg version 2 database), without any confirmed functional attributes.

### Joint Assessment of Host and Viral Factors as Independent Correlates of CD4:CD8 Ratio

During the 3- to 24-month period after EDI, geography, HLA-A*74:01 and low VL (<10,000 RNA copies/mL) were the major correlates of CD4:CD8 ratio >1.0, with adjusted *P*-values between <0.0001 and 0.048 (**Table 4**). Two potential confounders (age and sex) observed in healthy subjects (Amadori et al., 1995) had no obvious impact on CD4:CD8 ratio >1.0 in this cohort (adjusted *P* > 0.43), whereas low VL (<10,000 copies/ml) and HLA-A*74:01 were independent predictors of healthy CD4:CD8 ratio (OR > 2.0 in all tests), with further confirmation by an alternative model in which geography was replaced by viral subtype as a covariate (**Table 5**). By excluding three subjects with missing data (viral sequencing failed), the alternative model revealed that HIV-1 subtype A1 and infrequent subtypes (not A1 and not C) were positively associated with CD4:CD8 ratio >1.0 (adjusted OR = 1.74 and 1.89, *P* = 0.042 and 0.045, respectively, when compared with subtype C). Further refinement for the infrequent HIV-1 subtypes was not feasible, as neither subtype D (*n* = 75) nor recombinant forms (*n* < 20) were common enough to allow separate models.

**Table 4:** Host and viral factors as independent correlates of immunologic health (average CD4:CD8 ratio >1.0) in early HIV-1 infection.

	Ratio >1.0 during the 3- to 24-month intervals^a^			
Factors in the model^b^	*n*	OR^b^	95% CI	Adjusted *P*
Age > 40 years^c^	75	1.14	0.61–2.15	0.678
Female sex^c^	187	1.21	0.75–1.93	0.436
Region (Zambia)	196	0.61	0.37–1.00	0.048
HLA-A*74:01	64	2.07	1.14–3.78	0.017
Low VL (<10,000 RNA copies/mL)	142	2.71	1.67–4.39	<0.0001
High VL (>100,000)	92	0.53	0.24–1.17	0.117

*^a^Overall area under the curve (AUC) = 0.70 and *P* < 0.0001 for the model. Estimates of odds ratio (OR) and 95% CI have been adjusted for all factors in the model.*

*^b^Reference groups for several multi-entry factors are listed in **Table 3**.*

*^c^Age and sex have been associated with CD4:CD8 ratio in healthy subjects (Amadori et al., 1995).*

**Table 5:** An alternative model for assessing correlates of immunologic health (mean CD4:CD8 ratio >1.0) in 496 HIV-1 SCs.

		Mean CD4:CD8 ratio >1.0 in the 3–24 months period		
Factors in the joint model^c^	Subjects	OR^d^	95% CI^d^	Adjusted *P*
Age > 40 years	75	1.13	0.60–2.13	0.704
Female sex	185	1.15	0.72–1.84	0.569
HLA-A*74:01	63	2.01	1.09–3.68	0.025
HIV-1 subtype A1	183	1.74	1.02–2.95	0.042
HIV-1 subtype C	215	1.00	–	–
Other HIV-1 subtypes (not A1 or C)	98	1.89	1.02–3.50	0.045
Low VL (<10,000)	140	2.62	1.61–4.26	<0.0001
High VL (>100,000)	91	0.52	0.23–1.15	0.106

^a^HIV-1 subtype replaces geography as a covariate; three subjects with missing information for viral subtype are excluded; one of them has HLA-A*74:01.

*^b^Overall AUC = 0.70, *P* < 0.0001 for the model.*

*^c^Reference groups for several multi-entry factors are listed in **Table 3**.*

*^d^Estimates of OR and 95% CI have been adjusted for all factors in the model.*

### AUC Estimates for Predicting Healthy CD4:CD8 Ratios

For the overall cohort of 499 SCs, host and viral factors had robust AUC estimates for predicting average CD4:CD8 ratios >1.0 during the 3- to 24-month period after EDI (AUC = 0.70, *P* < 0.0001; **Table 4**). In additional models that tested two major HIV-1 subtypes separately, host and viral factors had similar predictive value for subtype C (AUC = 0.70, *P* < 0.0001) and subtype A1 infection (AUC = 0.65, *P* < 0.001) (data not shown).

## Discussion

In line with our primary hypothesis, prospective data from 499 HIV-1 SCs did suggest that CD4:CD8 ratio in early (primary) infection has three main features. First, this ratio is relatively stable during the first 3 years of HIV-1 infection, in that regard resembling the set-point VL (Prentice et al., 2014b). Second, early CD4:CD8 ratio is predictive of subsequent disease progression: a favorable ratio (>1.0) is a clear sign of immunologic health that is strongly associated with a delayed course to severe CD4 deficiency. Third, CD4:CD8 ratio has a rather weak correlation with two conventional and extensively studied outcome measures (VL and CD4 slope). As a result, QTLs (i.e., HLA factors) associated with CD4:CD8 ratio are expected to differ starkly from the well-known QTLs already documented for VL and CD4 count (Amornkul et al., 2013; Peterson et al., 2013; Prentice et al., 2013).

Although CD4 depletion is an important manifestation of HIV-1 pathogenesis, exacerbation of immunologic health can be further attributable to persistent immune activation (expression of CD38 and HLA-DR) driven by viral antigens and microbial translocation (Jirillo et al., 1991; Savarino et al., 2000; Brenchley et al., 2006; Douek, 2007). T-cell exhaustion (expression of PD-1) is another trait in HIV-1 infection that has gained close attention (Day et al., 2006; Petrovas et al., 2006; Trautmann et al., 2006). As immunophenotyping becomes increasingly feasible in resource-poor nations (Karita et al., 2009), analyses of banked and newly collected samples should help further elucidate the relationships between CD4:CD8 ratio and other established correlates of T-cell function. These parameters of immunologic health can be gradually incorporated into the HIV-1 treatment continuum when the focus shifts from virologic suppression to immune recovery and management of comorbidities (Buggert et al., 2014; Serrano-Villar et al., 2014). Until then, evidence from earlier work based on general populations (Amadori et al., 1995; Ferreira et al., 2010) and our analyses of HLA class I genes in HIV-1-infected Africans can pave the way for studying CD4:CD8 ratio as a genetically modulated and clinically relevant trait.

A putative association between HLA-A*74:01 and a favorable CD4:CD8 ratio is rather consistent with previous reports based on analyses of HIV-1 acquisition, VL and/or CD4 count after HIV-1 infection (Koehler et al., 2010; Leslie et al., 2010; Tang et al., 2010; Lazaryan et al., 2011; Peterson et al., 2013). Genetic diversity in our cohort of native Africans enabled us to rule out the potential confounding by other HLA class I alleles, which is a critical step toward a definitive dissection of functional mechanisms. The description of three HIV-1-specific CTL epitopes in another African cohort (Matthews et al., 2011) already suggests that antigen presentation by HLA-A*74:01 can direct CTL responses to multiple antigens. Furthermore, unlike *HLA-B* and *HLA-C* alleles that also mediate innate immunity through interaction with natural killer (NK) cell receptors (Carrington et al., 2008), HLA-A*74:01 is unlikely to have a prominent role in innate immunity. Such distinction, if proved true, would effectively eliminate the need for considering A*74:01-driven NK cell function that is expected to be more generic than adaptive immune responses.

HLA-A*74:01 and its proxy (allele T of rs9468675) are mostly restricted to Africans (de Bakker et al., 2006). While further attention to A*74:01-restricted HIV-1 epitopes (Matthews et al., 2011) may ultimately uncover relevant mechanisms of immunologic health in subjects with HLA-A*74:01, other loci (beyond the *HLA-A* locus) are known to regulate CD8 T-lymphocyte function in populations of European ancestry (Cruz et al., 2006, 2008; Ferreira et al., 2010). Further examination of SNPs associated with CD4:CD8 ratio in healthy subjects can be helpful (Ferreira et al., 2010), especially since A*74:01-positive subjects (~15%) in this study (Supplementary Table S2) can only account for <50% of those with a favorable CD4:CD8 ratio (**Figure 2**).

In our previous studies that focused on VL and CD4 count (two conventional outcome measures) in HIV-1-infected Africans, HLA-A*74:01 was not recognized as a prominent factor in systematic evaluation of cross-sectional and longitudinal data (Amornkul et al., 2013; Prentice et al., 2013). Instead, HLA-B*18, B*45, B*53, B*57, and B*81 were implicated, often in a time-sensitive manner (Amornkul et al., 2013; Prentice et al., 2013). To further establish independent correlates of CD4:CD8 ratio, multivariable models conditioned on VL and CD4 slope did not obscure the association of HLA-A*74:01 with immunologic health, suggesting that individuals with HLA-A*74:01 might have certain unique immunologic traits that analyses of VL and CD4 count alone cannot reveal. In other words, HLA factors may operate in different immune pathways to impact the manifestations of HIV-1 infection.

Host genetics aside, correlates of CD4:CD8 ratio further included HIV-1 subtype, even when VL was retained as a covariate in the analytic model (**Table 5**). High-throughput deep sequencing may eventually facilitate a better understanding of viral characteristics (Haaland et al., 2013) that are important to immunologic health, as viruses from acute and early chronic phases of infection can be readily compared for replicative fitness (Claiborne et al., 2015; Yue et al., 2015).

Despite our emphasis on longitudinal data, our study did have two apparent limitations that are worth reiterating. First, CD4:CD8 ratio was rarely measured before acquisition of HIV-1 infection. For subjects with CD4:CD8 ratio ≤1.0 soon after HIV-1 infection, we were unable to determine if this was the result of rapid disease progression or low CD4:CD8 ratio before infection. Second, the opportunity for studying opportunistic infections and other AIDS-defining conditions was precluded by treatment guidelines. These limitations can be a recurring issue in follow-up studies as well. A revisit to earlier cohorts with prolonged follow-up without therapy may offer a feasible option for answering questions about AIDS-related outcomes.

Overall, our study represents the first comprehensive comparison of CD4:CD8 ratio with VL, CD4 trajectory and CD4 deficiency in an African cohort with frequent follow-up. It is evident that measurement of CD4:CD8 ratio can have added value for predicting subsequent disease progression, at least irrespective of other known factors (demographics, HIV-1 subtypes, etc.). Certain characteristics seen in subjects with a favorable CD4:CD8 ratio, including HLA variants, may offer valuable insights into the determinants or mechanisms of immunologic health in HIV-1 infection. Recent approaches to fine mapping of causal variants in HLA genes and neighboring loci have provided promising leads for follow-up studies (McLaren et al., 2012; Prentice et al., 2014a; Farh et al., 2015).

## Author Contributions

JT, MP, ES, OA, EK, AK, SL, SA, EH, RK, and JG designed the study. ES, OA, EK, AK, SL, and SA assembled the cohort and gathered clinical data. JT and RK supervised and reviewed genotyping. XL and JT managed and analyzed the data. All authors contributed to the writing and proof reading of this manuscript.

## Conflict of Interest Statement

The authors declare that the research was conducted in the absence of any commercial or financial relationships that could be construed as a potential conflict of interest.

## Footnotes

1 http://www.hiv.lanl.gov/content/immunology

2 http://1drv.ms/1l5zG1a

3 http://www.broadinstitute.org/pubs/finemapping/?q=data-portal

4 http://www.broadinstitute.org/mammals/haploreg/haploreg.php
