# Supplementary material for: CD4:CD8 lymphocyte ratio as a quantitative measure of immunologic health in HIV-1 infection: findings from an African cohort with prospective data
Source: Front Microbiol. 2015 Jul 1;6:670. doi: 10.3389/fmicb.2015.00670 (PMC4486831; doi:10.3389/fmicb.2015.00670)
Supplement: Supplementary file 1 [file Table_1.DOCX]

**List of Supplemental Materials**

**Table S1**. Characteristics of 499 HIV-1 seroconverters stratified by the average CD4:CD8 ratio in the 3- to 24-month intervals after infection.

**Table S2**. HLA class I variants as potential correlates of favorable CD4:CD8 ratio (>1.0) in 499 HIV-1 seroconverters.

**Table S1.** Characteristics of 499 HIV-1 seroconverters stratified by their average CD4:CD8 ratio in the 3- to 24-month intervals after infection.

| Characteristics | CD4:CD8 ≤1.0 (*N* = 394)^a^ | CD4:CD8 >1.0 (*N* = 105)^a^ | *P*^a^ |
| --- | --- | --- | --- |
| Age: mean ± SD (year) | 31.1 ± 8.4 | 32.4 ± 8.2 | 0.180 |
| Age ≥40 years: n (%) | 59 (15.0) | 16 (15.2) | 0.947 |
| Sex ratio (M/F) | 1.77 (252/142) | 1.33 (60/45) | 0.200 |
| Country^b^: no. (%) |  |  | <0.001 |
| Kenya | 87 (22.1) | 15 (14.3) | 0.079 |
| Rwanda | 56 (14.2) | 20 (19.1) | 0.221 |
| Uganda | 84 (21.3) | 41 (39.0) | <0.001 |
| Zambia | 167 (42.4) | 29 (27.6) | 0.006 |
| HIV-1 subtype: no. (%) |  |  | <0.012 |
| A1 | 135 (34.4) | 48 (46.6) | 0.022 |
| C | 184 (46.8) | 31 (30.1) | 0.002 |
| D | 57 (14.5) | 18 (17.5) | 0.454 |
| Others (B and recombinants)^c^ | 18 (4.6) | 8 (7.6) | 0.212 |
| HIV-1 viral load^d^: *n* (%) |  |  | <0.0001 |
| Low (<10,000) | 89 (22.6) | 53 (50.5) | <0.0001 |
| Medium (10,000-100,000) | 221 (56.1) | 44 (41.9) | 0.010 |
| High (>100,000) | 84 (21.3) | 8 (7.6) | 0.001 |
| HLA variants of interest: *n* (%) |  |  |  |
| A*74 (B*74:01) | 42 (10.7) | 22 (21.0) | 0.005 |
| B*18 (B*18:01)^e^ | 25 (6.4) | 9 (8.6) | 0.421 |
| B*44 (B*44:03 & B*44:15)^e^ | 38 (9.6) | 13 (12.5) | 0.411 |
| B*45 (B*45:01)^e^ | 69 (17.5) | 12 (11.4) | 0.133 |
| B*53 (B*53:01)^e^ | 76 (19.3) | 18 (17.1) | 0.618 |
| B*57 (mostly B*57:03)^e^ | 35 (8.9) | 11 (10.5) | 0.617 |
| B*81 (B*81:01)^e^ | 18 (4.6) | 7 (6.7) | 0.382 |

^a^ At least four visits per patient. Overall *P* values for three multi-entry variables are underlined.

^b^ Kenya, Rwanda and Uganda are considered as eastern Africa in terms of geography.

^c^ Including three subjects with missing information.

^d^ Geometric mean VL in the 3-24 months period when CD4:CD8 ratio is also measured in parallel.

^e^ Factors already highlighted in earlier analyses of VL and CD4 count.

**Table S2.** HLA class I variants as potential correlates of favorable CD4:CD8 ratios (>1.0) in 499 HIV-1 seroconverters.

| HLA variants^a^ | *n* | Odds ratio | 95% confidence interval | Adjsuted *P*^b^ | FDR (*q*) |
| --- | --- | --- | --- | --- | --- |
| A*01 | 62 | 0.72 | 0.36-1.43 | 0.353 | 0.849 |
| A*02 | 174 | 0.82 | 0.51-1.30 | 0.393 | 0.849 |
| A*03 | 45 | 0.86 | 0.40-1.87 | 0.706 | 0.867 |
| A*23 | 75 | 0.79 | 0.40-1.54 | 0.485 | 0.849 |
| A*29 | 47 | 1.69 | 0.84-3.38 | 0.141 | 0.849 |
| A*30 | 177 | 0.89 | 0.56-1.42 | 0.626 | 0.849 |
| A*33 | 25 | 0.35 | 0.08-1.50 | 0.157 | 0.849 |
| A*34 | 36 | 1.62 | 0.74-3.53 | 0.225 | 0.849 |
| A*36 | 41 | 0.79 | 0.34-1.86 | 0.595 | 0.849 |
| A*66 | 32 | 0.87 | 0.34-2.20 | 0.765 | 0.867 |
| A*68 | 121 | 1.50 | 0.92-2.42 | 0.102 | 0.849 |
| A*68:02^c^ | 103 | 1.10 | 0.65-1.86 | 0.725 | − |
| A*74 | 64 | 2.29 | 1.28-4.08 | 0.005 | 0.172 |
| B*07 | 66 | 1.03 | 0.55-1.94 | 0.925 | 0.953 |
| B*14 | 46 | 1.32 | 0.64-2.73 | 0.453 | 0.849 |
| B*15 | 157 | 0.85 | 0.53-1.38 | 0.522 | 0.849 |
| B*18 | 34 | 1.38 | 0.62-3.10 | 0.428 | 0.849 |
| B*35 | 28 | 0.65 | 0.22-1.93 | 0.434 | 0.849 |
| B*42 | 66 | 1.22 | 0.66-2.27 | 0.521 | 0.849 |
| B*44 | 51 | 1.51 | 0.76-3.00 | 0.239 | 0.849 |
| B*45 | 81 | 0.64 | 0.33-1.24 | 0.183 | 0.849 |
| B*49 | 38 | 0.42 | 0.16-1.12 | 0.085 | 0.849 |
| B*53 | 94 | 0.88 | 0.49-1.55 | 0.649 | 0.849 |
| B*57 | 46 | 1.22 | 0.59-2.52 | 0.585 | 0.849 |
| B*58 | 126 | 0.92 | 0.56-1.53 | 0.755 | 0.867 |
| B*58:01^c^ | 56 | 1.40 | 0.74-2.67 | 0.302 | − |
| B*58:02^c^ | 72 | 0.68 | 0.35-1.32 | 0.251 | − |
| B*81 | 25 | 1.46 | 0.58-3.64 | 0.419 | 0.849 |
| C*02 | 91 | 1.15 | 0.65-2.01 | 0.633 | 0.849 |
| C*03 | 72 | 0.82 | 0.43-1.56 | 0.538 | 0.849 |
| C*04 | 158 | 0.84 | 0.52-1.36 | 0.478 | 0.849 |
| C*06 | 146 | 1.03 | 0.64-1.65 | 0.919 | 0.953 |
| C*07 | 179 | 0.95 | 0.60-1.50 | 0.835 | 0.915 |
| C*08 | 63 | 1.37 | 0.73-2.55 | 0.327 | 0.849 |
| C*16 | 68 | 0.89 | 0.46-1.71 | 0.722 | 0.867 |
| C*17 | 80 | 1.00 | 0.55-1.81 | 1.000 | 1.000 |
| C*18 | 42 | 1.30 | 0.61-2.78 | 0.501 | 0.849 |

^a^ Frequencies ≥5.0% in the entire cohort. **B*18, B*45, B*53, B*57 and B*81 have been associated with viral load and/or CD4 count in earlier analyses (see text).**

^b^ Age, sex, and geography are treated as covariates in logistic regression models.

^c^ Secondary analyses for distinct 4-digit alleles relevant to Africans.
